# Study on the Fermented Grain Characteristics and Volatile Flavor Substances during the Tuqu Fermentation of Hunan Light-Flavor Baijiu

**DOI:** 10.3390/foods13060899

**Published:** 2024-03-15

**Authors:** Jie Xu, Ting Zhang, Huitai Chen, Yijie Dai, Zongjun Li, Jia He, Rongfang Ju, Aixiang Hou

**Affiliations:** 1 College of Food Science and Technology, Hunan Agricultural University, Changsha 410128, China; 18798312569@163.com (J.X.); zhangting9909@163.com (T.Z.); rehjia189@163.com (J.H.); 15284676937@163.com (R.J.); 2 Key Laboratory of Food Science and Biotechnology of Hunan Province, Changsha 410128, China; 3 Hunan Guoyuan Liquor Co., Ltd., Miluo 414400, China; 18673187905@163.com; 4 School of Biology and Environmental Engineering, Guiyang University, Guiyang 550005, China; daiyijie866@163.com (Y.D.); hnlizongjun@163.com (Z.L.)

**Keywords:** light-flavor Baijiu, fermented grains, bacteria, fungi, volatile flavor substances

## Abstract

The present study employed Hunan local Tuqu for fermentation and investigated the physicochemical properties, microbial community composition, and volatile flavor compounds of the fermented grains, as well as the correlation between the physicochemical indicators and the microbial community. The findings reveal that the activities of α-amylase and glucoamylase were highest during the initial stages of the fermentation process. The acid protease activity increased to 30.6 U/g on the second day and then decreased. Cellulose and lipase activities both showed an increasing trend. The moisture content increased sharply to 73.41% and then remained relatively stable. The acidity was highest on the eighth day. Fifty genera of bacteria and twenty-two genera of fungi were detected. *Lactobacillus* was dominant among bacteria, and *Saccharomyces* was dominant among fungi. A correlation analysis showed that there were positive correlations between moisture, acidity, cellulose, lipase activities and *Lactobacillus*, and there were positive correlations between moisture content, acidity, cellulase activity, acidic protease activity and *Saccharomyces*. A total of 46 volatile flavor compounds were detected, of which 6 alcohols and 14 esters constituted the major portion, and 9 key flavor compounds with an ROAV > 1 were identified throughout the fermentation process. Isoamyl acetate had the highest ROAV and made the greatest contribution to the flavor.

## 1. Introduction

As one of the oldest distilled liquors in the world, Chinese Baijiu has a history of thousands of years [1]. In the process of development, due to the brewing of raw materials, fermentation strains, brewing techniques, and regional differences, 12 types of Baijiu, such as sauce-flavor, strong-flavor, light-flavor, and rice-flavor Baijiu, have gradually developed [2]. The liquor market in Hunan province is highly inclusive, open, and of high consumption grade, but the competitiveness of real estate liquor is weak and the market share is low. The annual sales of liquor are nearly 30 billion yuan, but the consumption of real estate liquor is only more than 5 billion yuan, accounting for about one sixth of the market share. Therefore, in order to improve the competitiveness and market value of local liquor, it is necessary to study a liquor with local Hunan characteristics.

As one of the oldest and most popular types of Baijiu, light-flavor Baijiu, is pure, sweet, mild, natural, and has a fresh aftertaste [3]. It can be divided into Daqu, Xiaoqu, and Fuqu light-flavor Baijiu according to the type of starter culture [4]. Microbial starter cultures play a very important role in the fermentation process of Baijiu. It has been shown that bacteria have an important function in the production of enzymes and aromas during the fermentation of Baijiu. The flavor substances produced by the metabolism of bacteria play a decisive role in the aroma and quality of the liquor [5]. Fungi play a key regulatory role in the formation of flavor compounds and in ensuring the consistent food quality of fermented foods [6]. Microorganisms of fermented grains interact with moisture, acidity, and enzyme activity during fermentation, so it was necessary to investigate their interactions during fermentation. In recent years, research on light-flavor Baijiu has focused on the influence of microbial flora, the structure of functional strains, and flavor substances on the style of Baijiu [7,8,9]. However, the physicochemical properties, microbial communities, and dynamics of the flavor compounds in fermented grains are still not fully understood.

During the fermentation of Baijiu, the microbial composition and environmental factors can have a great influence on the community succession during the fermentation of Baijiu. Microbial interactions affect the metabolic behavior of microorganisms involved in food fermentation and, consequently, changes in volatile flavor substances. Therefore, exploring the physicochemical properties, microbial communities, and volatile flavor compounds of fermentation grains could not only reveal the changing rules of the physicochemical, microbial community, and volatile flavor substances of the fermentation process, but also provide an important reference for controlling the quality of clear-flavored original wine from the perspective of fermentation. In this study, the microbial flora composition of different fermentation stages was analyzed by using Hunan Yueyang and Liuyang Tuqu in the mixed fermentation of light-flavor Baijiu. The moisture content, acidity, associated enzyme activity, and major volatile flavor substances were determined, and correlations between microorganisms and the moisture, acidity, and enzyme activity during fermentation were analyzed. The aim was to investigate the evolution of the microbial flora, the variations in moisture, acidity, enzymatic activity, and other physicochemical characteristics, and the formation of volatile flavor substances in the Tuqu fermentation process of Hunan light-flavored Baijiu, in order to provide data reference and theoretical guidance for the development of Hunan characteristic light-flavor Baijiu.

## 2. Materials and Methods

### 2.1. Sample Collection

**Figure 1:**
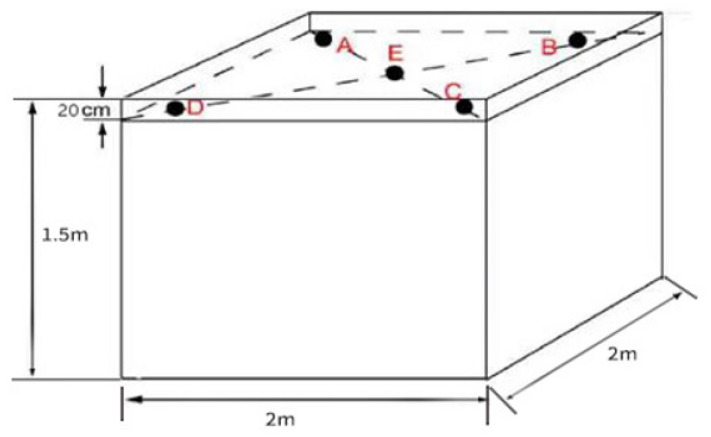
Sampling sites of the fermented grain samples.

All samples for this study were collected from the top of the cellar at 0 days, 2 days, 4 days, 6 days, 8 days, and 10 days of fermentation. The five-point sampling method was used for sampling. The specific sampling points are shown in Figure 1. The samples collected at each point were mixed and stored in a liquid nitrogen jar.

### 2.2. The Brewing Process of the Tuqu Fermentation of Hunan Light-Flavor Baijiu

**Figure 2:**
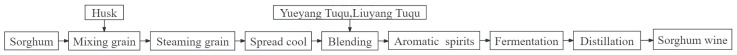
The brewing process of Hunan light-flavor Baijiu.

The technological process of the Tuqu fermentation of Hunan light-flavor Baijiu in this study is shown in Figure 2.

### 2.3. Major Reagents and Instruments

Concentrated sulfuric acid (analytical and pure): Nanjing Sheng Qing He Chemical Co., Ltd. (Nanjing, China); toluene (analytical purity): Wuxi Yashang Chemical Co., Ltd. (Yizing, China); A-amylase (A-AL) kit, acid protease (ACP) kit, glucoamylase (Gluco) kit, cellulase (CL) kit, and lipase (LPS) kit: Suzhou Kemin Biotechnology Co., Ltd. (Suzhou, China); constant temperature oscillation water bath: Hunan Jinnan Instrument Manufacturing Co., Ltd. (Jinan, China); MD enzyme standard instrument: Meigu Molecular Instruments (Shanghai, China) Co., Ltd.; CJJ78-1 Magnetic Stirrer: Wuhan Glamour Testing Equipment Co., Ltd. (Wuhan, China); H1200R high-speed frozen centrifuge: Shanghai Precision Instrument Company (Shanghai, China); F6/10 handheld high-speed homogenizer: Shanghai Jingxin Industrial Development Co., Ltd. (Shanghai, China); XUEKE ice-making machine: Beijing Zhongxing Baihui Technology Co., Ltd. (Beijing, China); ZYS-50B wise copy energy measuring water meter: XiangYi Balance Instrument Co., Ltd. (Xiangtan, China).

### 2.4. Determination of Physical and Chemical Indicators

The enzyme activity was determined according to the kit instructions. The moisture was determined using an intelligent moisture tester. The acidity was determined according to the “DB34/T2264-2014” [10] fermentation method for solid fermented grains, and the acid–base neutralization titration method was used for the determination.

### 2.5. DNA Extraction, Polymerase Chain Reaction (PCR) Amplification, and Sequencing

#### 2.5.1. DNA Extraction

The total microbial genomic DNA samples were extracted using the DNeasy PowerSoil Kit (QIAGEN, Inc., Venlo, The Netherlands) following the manufacturer’s instructions and stored at −20 °C prior to further analysis. The quantity and quality of the extracted DNAs were measured using a NanoDrop ND-1000 spectrophotometer (Thermo Fisher Scientific, Waltham, MA, USA) and agarose gel electrophoresis, respectively.

#### 2.5.2. 16S rRNA and ITS Gene Amplicon Sequencing

PCR amplification of the bacterial 16S rRNA gene V3-V4 region was performed using the forwards primer 338F (ACTCCTACGGGAGGCAGCA) and the reverse primer 806R (GGACTACHVGGGTWTCTAAT). PCR amplification of fungal internal transcribed spacer (ITS) regions was performed with the primers ITS3 (GCATCGATGAAGAACGCAGC) andITS4 (TCCTCCGCTTATTGATATGC). Sample-specific 7-bp barcodes were incorporated into the primers for multiplex sequencing. The PCR components contained 5 μL of Q5 reaction buffer (5×), 5 μL of Q5 HighFidelity GC buffer (5×), 0.25 μL of Q5 High-Fidelity DNA Polymerase (5 U/μL), 2 μL (2.5 mM) of dNTPs, 1 μL (10 µM) of each forward and reverse primer, 2 μL of DNA template, and 8.75 μL of ddH2O. Thermal cycling consisted of initial denaturation at 98 °C for 2 min, followed by 25 cycles consisting of denaturation at 98 °C for 15 s, annealing at 55 °C for 30 s, and extension at 72 °C for 30 s, with a final extension of 5 min at 72 °C. PCR amplicons were purified with Agencourt AMPure Beads (Beckman Coulter, Indianapolis, IN, USA) and quantified using the PicoGreen dsDNA Assay Kit (Invitrogen, Carlsbad, CA, USA). After the individual quantification step, amplicons were pooled in equal amounts, and paired-end 2 × 300 bp sequencing was performed using the Illumina MiSeq platform with MiSeq Reagent Kit v3 at Shanghai Personal Biotechnology Co., Ltd. (Shanghai, China).

### 2.6. Determination of Volatile Flavor Substances

Headspace solid phase microextraction was employed to extract VOCs from the fermented grain samples. The detailed method was carried out as follows: in a headspace vial with a capacity of 20 mL equipped with a magnetic stirrer, 3 g of the fermented grain sample, 2 g of sodium chloride, 20 μL of amyl acetate (0.5275 mg/mL) as an internal standard solution, and 4 mL of boiled ultra-pure water were combined. The top of the glass bottle was sealed with a silicon diaphragm to create a sealed headspace environment. The headspace glass bottle was then heated to 50 °C, and the tea sample was stirred at a speed of 1100 rpm using the magnetic stirrer. After a 10 min pre-balance period, a 50/30 μm DVB/CAR/PDMS fiber from American Supelco was inserted into the headspace vial to extract the VOCs. The extraction process lasted for 45 min. Subsequently, the fiber was desorbed for five minutes at the injection port of the GC–MS, operating in splitless injection mode at 250 °C.

Head space solid phase microextraction (HS-SPME):

The gas chromatography conditions were as follows: column, Hp-5 ms, ultra-inert (−60~325 °C) 30 m × 250 mm × 0.25 μm; injection temperature, 250 °C in splitless mode; heating procedure, a starting temperature of 50 °C, maintained for 2 min, increased to 230 °C at 4 °C/min and maintained for 5 min; and column carrier gas, helium at a constant flow rate of 1 mL/min.

The mass spectrometric conditions were as follows: ionization source, electron impact (EI); ion source temperature, 230 °C; EI energy, 70 eV; quadrupole temperature, 150 °C; scan mode, constant pressure full scan; and mass spectrometric scan range, 30–600 amu. We compared the mass spectrometry information of each chromatographic peak obtained from GC-MS analysis with the reference compounds in the NIST 17 library to identify unknown compounds [11]. To calculate the retention index (RI) for each peak, the retention time (RT) of *n*-alkanes (C7~C21) under the same GC–MS conditions was utilized. The identification of VOCs was accomplished by comparing the calculated RI values and the mass spectra of all detected metabolites with those present in the NIST 17 library. For the quantification of VOCs, the peak area ratio of the VOCs to the internal standard ethyl caprate was multiplied by the concentration of ethyl caprate.

### 2.7. Analysis of Relative Odor Activity Value

The relative odor activity value (ROAV) is a method used to identify key volatile compounds in samples, and VOCs with an ROAV value greater than or equal to 1 are considered key volatile compounds. VOCs with an ROAV value ranging from 0.1 to <1 have a modifying effect on the overall flavor of the sample. The ROAV calculation method was based on the ratio of a volatile component’s odor activity value (OAV) to the greatest odor activity value (OAVmax) among the volatile compounds in the Tuqu fermentation of Hunan light-flavor Baijiu [12], and the thresholds were obtained by finding the threshold of perception of each volatile compound in the water based on [13].

### 2.8. Statistical Analysis

Experimental data were analyzed using the Origin 2018 statistical program. Correlation analysis was performed using SPSS v. 22.0 software. The raw data generated by the Illumina NovaSeq250 sequencing platform were used for sequence data analysis using the QIIME and R package (v3.2.0). The community composition and heatmaps based on the R language were generated using the Illumina NovaSeq250 sequencing platform.

## 3. Results and Discussion

### 3.1. Variation Rules of Enzyme Activity during the Tuqu Fermentation of Hunan Light-Flavor Baijiu

In the Baijiu brewing process, enzymes, as key substances in Baijiu fermentation, have various functions, such as degrading fermented raw material particles, promoting microbial reproduction and catalyzing biochemical reactions to produce aroma substances. The variations in enzyme activities during the Tuqu fermentation of Hunan light-flavor Baijiu are shown in Table 1.

As shown in Table 1, the α-amylase and glycolytic enzyme activities decreased during the fermentation process, and the α-amylase enzyme activity decreased from 28.1 U/g to 3.8 U/g. The acidity and alcoholic content increase during the fermentation process, which inhibits α-amylase activity [14]. The glucoamylase activity decreased from 1027.1 U/g to 766.7 U/g. *Aspergillus* can produce glucoamylase at the beginning of fermentation, which makes raw starch material decompose rapidly. As fermentation progresses, fungi such as *Aspergillus* decline gradually, and glucoamylase activity also decreases gradually [15]. Acid protease activity was highest on the second day of fermentation. Fungi such as *Aspergillus*, *Penicillium*, and *Rhizopus* can produce acid proteases. In the early stages of fermentation, there was sufficient oxygen and nutrients in the fermented grains, which promoted the proliferation of microorganisms; therefore, the activity of acid protease was increased. Cellulase activity and lipase activity showed an overall increasing trend, reaching peaks of 668.7 U/g and 36.4 U/g on day 10, respectively. Cellulase can act on fibrous raw materials, while lipase can act on ester bonds, making full use of the raw materials.

### 3.2. Variation in Moisture and Acidity during the Tuqu Fermentation Process of Hunan Light-Flavor Baijiu

**Figure 3:**
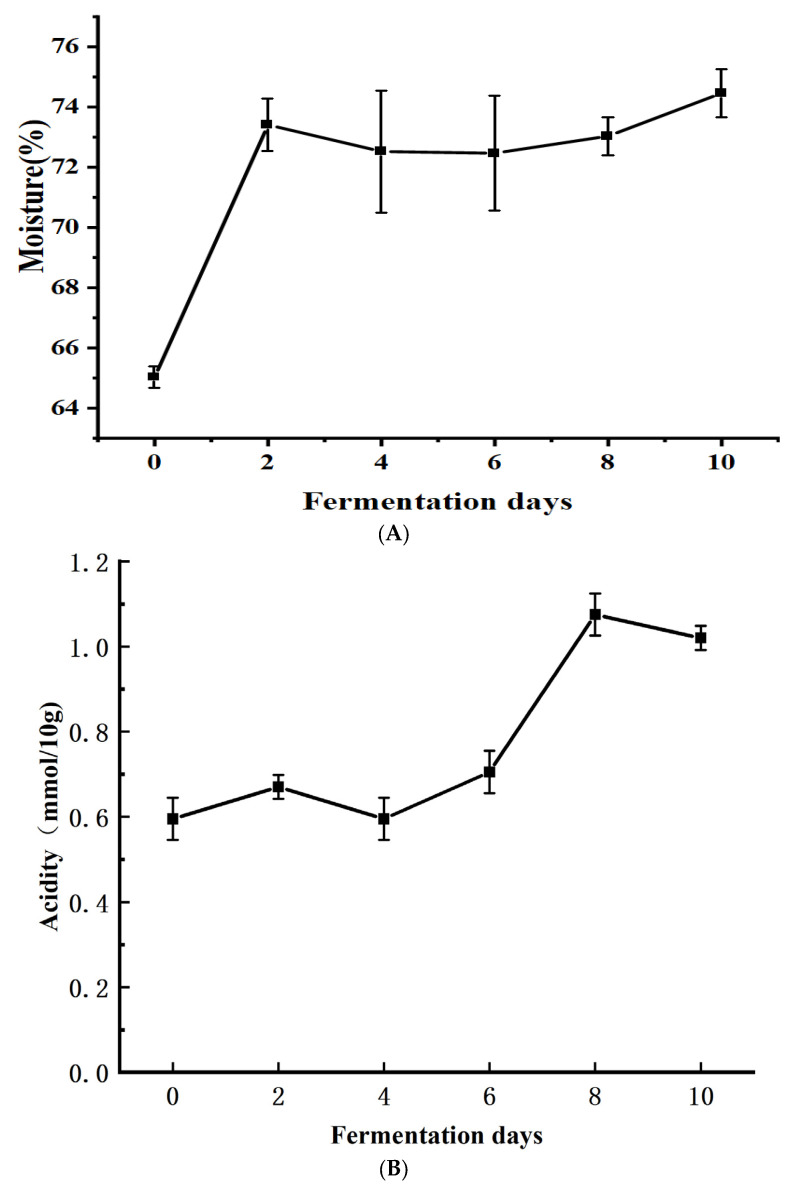
Variation of moisture and acidity with fermentation days during the Toqu fermentation of Hunan light-flavor Baijiu ((**A**): moisture; (**B**): acidity).

As shown in Figure 3A, from the initial stage of fermentation, the moisture content increased sharply from 65.04% to 73.41% and remained stable. During the prefermentation period, the source of moisture is mainly the water added during production as well as the water produced by the interaction between microorganisms [3]. During the fermentation process, volatile flavor substances such as ethanol, acids, and esters are produced along with water, thus increasing the moisture content of the fermented grains [16]. There was a slight decrease in moisture content at 2–10 days, and then it slowly increased again. This was due to the effect of gravity; the water flowed to the bottom of the cellar, becoming the main component of the yellow water, resulting in a decrease in moisture. During the fermentation process, with the progress of fermentation, yeast and other microorganisms conducted ethanol fermentation to produce free water, so the water of the fermented grains increased.

The acid in the fermented grains is mainly produced by microbial metabolism and is an important flavor substance in the Baijiu body, which can play a role in flavor enhancement, and the right amount of acid can increase the after-taste of liquor and make the Baijiu body fuller [17]. As seen in Figure 3B, the overall acidity showed an upward trend. As fermentation proceeded, anaerobic and partly anaerobic bacteria proliferated to produce a variety of organic acids, such as lactic acid and acetic acid, to increase the acidity.

### 3.3. Variations in the Structure of Bacteria during the Tuqu Fermentation of Hunan Light-Flavor Baijiu

#### 3.3.1. Bacterial Alpha Diversity Analysis of the Fermented Grains

**Figure 4:**
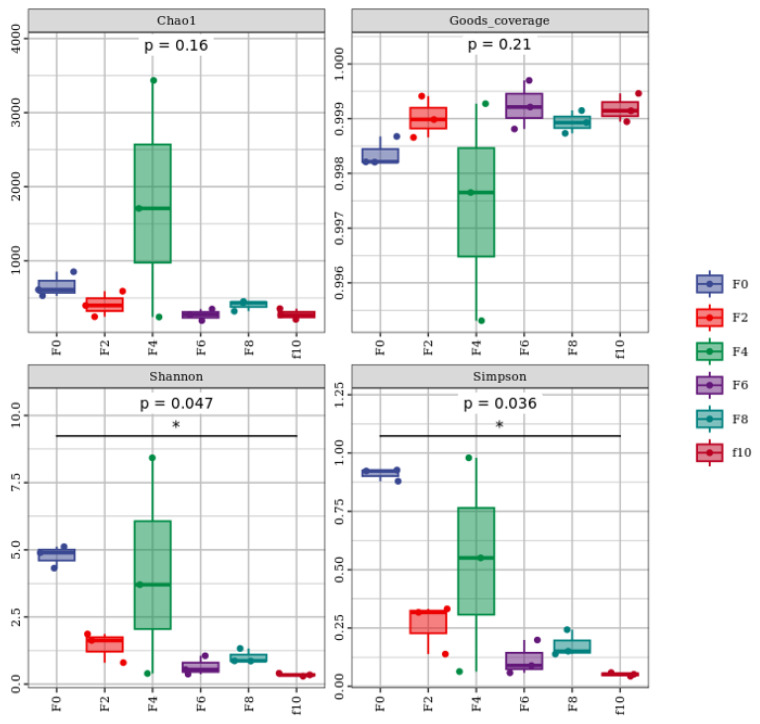
Alpha diversity analysis of bacteria in the Tuqu fermentation process of Hunan light-flavor Baijiu. * is a marker indicating significance.

The complexity of the species diversity of the studied samples was analyzed by calculating α-diversity indices. In α-diversity analysis, the Chao 1 index is mainly related to the abundance of the samples, and the larger the Chao 1 index is, the higher the abundance of the samples. The Shannon index is mainly related to the diversity of the sample, which reflects not only the richness but also the evenness of the species; the larger the Shannon index is, the higher the diversity [7]. As shown in Figure 4, the Chao1 index of F4 was higher than that of the other sample groups, which indicates that the total number of bacterial species in the F4 group was higher than that in the other sample groups. The Shannon index was higher in the F0 group than in the other sample groups, which indicates that the diversity of bacterial species was higher in the F0 group than in the other sample groups. The Shannon index decreased continuously as the fermentation proceeded, which indicates that the unfavorable conditions of low oxygen, low pH values, and high ethanol caused many bacteria to be inhibited. The coverage of all samples was greater than 0.996, and the *p* value of the Chao1 index was 0.069, which indicates that the library coverage of each sample was high, the sequences were basically completely sequenced, and the sequencing results were reliable.

#### 3.3.2. Analysis of the Bacterial Community Structure of the Fermented Grains

**Figure 5:**
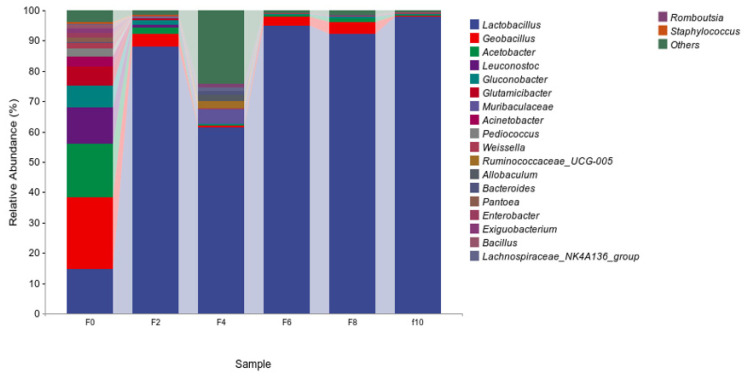
Community structure distribution of bacteria based on genus level during the Tuqu fermentation of Hunan light-flavor Baijiu.

Figure 5 shows the relative abundance of species in the top 20. As seen from the figure, the bacterial flora in the fermented grains at the genus level consisted mainly of *Lactobacillus*, *Geobacillus*, *Pediococcus*, and *Acetobacter*. As the fermentation progressed, *Lactobacillus* accounted for over 60% of the total sequence readings at 2–10 days, and a peak value of 97.97% was observed at the tenth day. The acidity and alcoholic strength increased, which caused the extinction of many bacteria; therefore, *Lactobacillus* became the dominant bacteria. *Lactobacillus* could affect the growth of other microorganisms by producing antagonistic substances such as bacteriocins and by competing with other microorganisms for substrates [18,19]. These were the dominant bacterial genera in the fermented grains. In addition, *Lactobacillus* were identified as the most abundant bacteria during the fermentation of the fermented grains, which was consistent with the results revealed in previous studies [20]. The abundances of *Geobacillus* and *Acetobacter* were highest, reaching peaks of 23.75% and 17.58% at 0 days, respectively, and then decreasing gradually. During the fermentation of the liquor, *Bacillus* can produce some flavor synthesis precursors in addition to the direct flavor substances obtained from their own fermentation. Complex flavor substances are further synthesized by a number of chemical or biological interactions [21]. *Acetobacter* are specialized aerobic bacteria that play an important role in the aroma presentation of Baijiu. There are abundant bacteria, such as *Acetobacter*, in the environment that can break down different types of sugars and alcohols into organic acids in oxygen [22].

#### 3.3.3. Heat Map Analysis of the Fermented Grain Bacterial Community Succession

**Figure 6:**
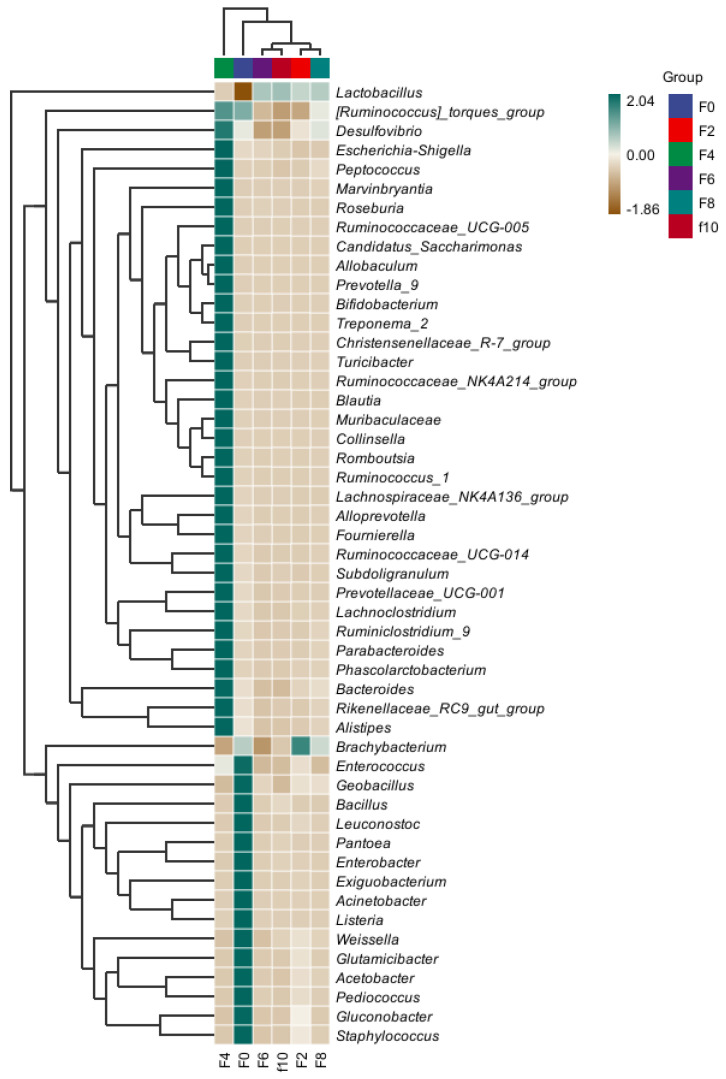
Heat map of the genus-based level of bacteria in the Tuqu fermentation process of Hunan light-flavor Baijiu.

Figure 6 shows a heat map of the bacterial composition during the Tuqu fermentation of Hunan light-flavor Baijiu, where a color closer to green indicates a greater relative abundance of the species, and a color closer to brown indicates a smaller relative abundance of the genus [23]. As shown in Figure 6, the bacterial diversity and abundance in the F0 and F4 stages were more diverse, including bacteria such as *Bacillus* and *Weissella*. As fermentation progressed, the diversity and abundance in the fermented grains decreased rapidly. It is possible that the increase in acidity as well as the ethanol content inhibited the growth of these bacteria. *Lactobacillus* became the dominant bacterium in the fermented grains. *Lactobacillus* is a class of anaerobic or partly anaerobic bacteria that can ferment sugars to produce lactic acid and provide amino acids and various vitamins and bacteriocins for the growth and reproduction of other microorganisms. *Lactobacillus* plays an important role in the microecological environment of brewing [24].

### 3.4. Variations in the Structure of Fungi during the Tuqu Fermentation of Hunan Light-Flavor Baijiu

#### 3.4.1. Fungi Alpha Diversity Analysis

**Figure 7:**
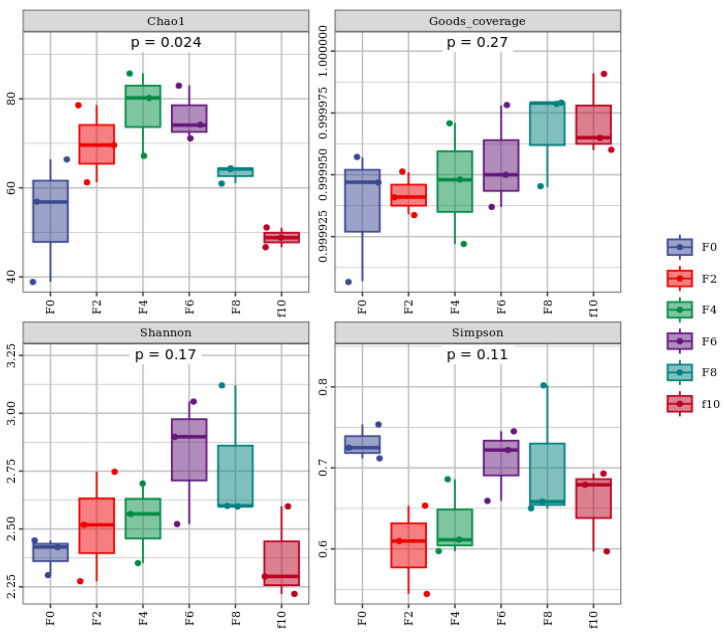
Alpha diversity analysis of fungi in the Tuqu fermentation process of Hunan light-flavor Baijiu.

From the α-diversity index in Figure 7, it can be seen that the coverage of each group of samples was greater than 0.999, and the *p* value of the Chao1 index was 0.014, which indicates that the sequencing depth was sufficient. The sequences in the samples were basically detected completely, and the results were true and reliable for subsequent analysis. The Chao1 index in F4 was the highest, which indicates that the total number of fungal species was the highest in the sample groups. The Shannon index of the F6 group was the highest, which indicates that the diversity of fungal species in the F6 group was the highest in the samples.

#### 3.4.2. Analysis of the Fermented Grain Fungi Community Structure

**Figure 8:**
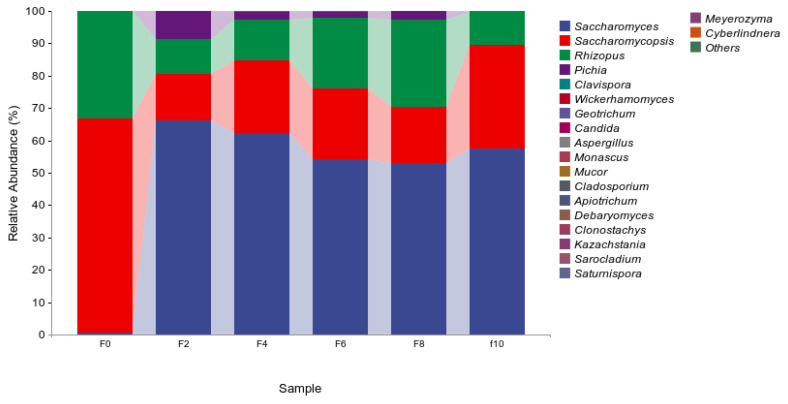
Community structure distribution of Fungi based on genus level during the Tuqu fermentation of Hunan light-flavor Baijiu.

Figure 8 shows the top 20 species in terms of relative abundance. As seen from the figure, *Saccharomyces*, *Saccharomycopsis*, *Rhizopus*, and *Pichia* were the four main fungal genera. Among them, *Pichia* had the highest abundance on the second day, which gradually decreased as fermentation progressed. It has been shown that *Pichia* plays a major role in the production of aromas in Baijiu brewing and has the ability to produce liquor metabolism and at this stage, yeasts and bacteria proliferate and produce flavor substances from the available sugars and other nutrients [25]. This is consistent with previous research [26]. *Saccharomyces cerevisiae* is the main functional bacterium in the fermentation process of liquor, which not only has a high fermentation capacity but also produces esters and plays an important role in the fullness of the liquor [27]. During the prefermentation period, *Saccharomycopsis* and *Rhizopus* were the main fungi, and the relative abundance decreased as fermentation progressed. It might be that the increased concentration of organic acids and alcohols disrupted the growth environment. As fermentation proceeded, *Saccharomyces* was the main fungus, reaching a peak of 66.20% on the second day. *Saccharomyces cerevisiae* can grow and reproduce in an anaerobic environment and is also tolerant to ethanol [2].

#### 3.4.3. Heat Map Analysis of Fungal Community Succession Based on the Species Taxonomic Genus Level

**Figure 9:**
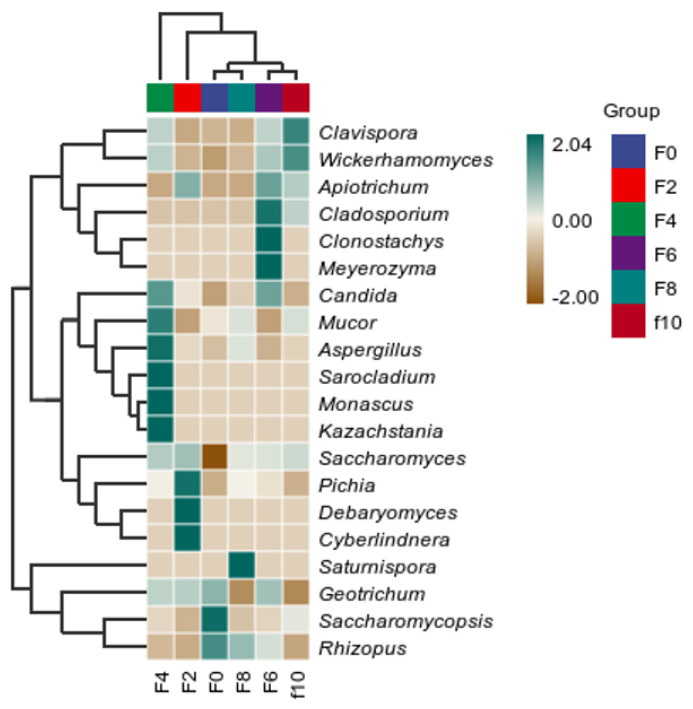
Heat map of the genus-based level of Fungi in the Tuqu fermentation process of Hunan light-flavor Baijiu.

Figure 9 shows a heatmap of the fungal composition during the fermentation of Tuqu-fermented grains of Hunan light-flavor Baijiu. A color closer to green represents a higher abundance, and a color closer to brown represents a lower abundance. As shown in the graph, as fermentation progressed, there were different dominant fungi at different fermentation stages, indicating a rich microbial structure. The main dominant fungi at the F0 stage were *Trichosporon*, *Saccharomycopsis* and *Rhizopus*. The main dominant fungi at the F2 stage were *Pichia*, *Debaryomyces*, and *Cyberlindnera*. The main fungal genera at the F4 stage were *Mucor*, *Aspergillus*, *Kazachstania*, *Monascus,* and *Sarociadium*. The main dominant fungi at the F6 stage were *Meyerozyma*, *Cladosporium*, and *Clonostachys*. The main dominant fungi at the F8 stage were *Rhizopus* and *Satumispora*, and the main dominant fungi at the F10 stage were *Clavispora* and *Wickerhamomyces*. These fungi are important fungal genera in the fermentation process of Baijiu. Among them, *Wickerhamomyces* and *Aspergillus* play a key role in fermentation. *Aspergillus* can produce a variety of enzymes and plays a positive role in regulating the *saccharification*, *esterification*, and liquefaction power of Daqu and fermented grains. In addition, it can metabolize organic acids and fatty acids, which can lead to the production of aromatic esters that enhance and improve the flavor of Baijiu [28]. *Wickerhamomyces* is an important aromatic yeast with a high acidity tolerance and a high ester production capacity, which can impart a rich aroma to the liquor [29].

### 3.5. Correlation Analysis of Microbial and Physicochemical System Indicators in the Tuqu Fermentation Process of Hunan Light-Flavor Baijiu

#### 3.5.1. Correlation Analysis of Bacteria at the Genus Level and Physicochemical Indicators

**Figure 10:**
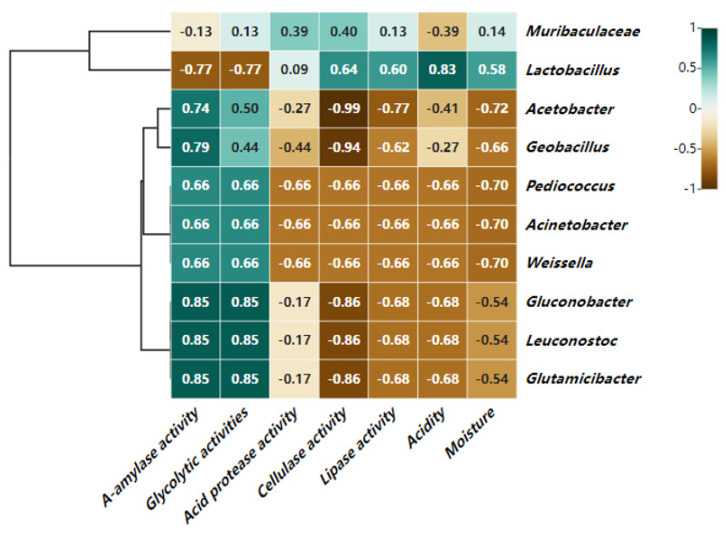
Heat map of correlation between bacterial microorganisms and physicochemical indicators based on genus level.

Spearman correlation analysis of physicochemical indicators and bacteria that were the top 10 bacterial genera in the fermented grains was performed using SPSS software. As shown in Figure 10 The closer the color was to green, the stronger the correlation was, and the closer it was to brown, the weaker the correlation was. As shown in the graph, *Lactobacillus* was positively correlated with moisture content, acidity, cellulase activity, and lipase activity, which suggests that the acidity and moisture were the main factors affecting microbial succession, which is consistent with previous research on the light-flavor liquor [30] indicating that *Lactobacillus* might produce cellulase and lipase [31]. *Lactobacillus* has a strong ability to metabolize carbohydrates and produce acid, providing a source of carbon for other microorganisms. It is a precursor to the formation of flavor during the fermentation of liquor [32]. The other three genera, *Geobacillus*, *Pediococcus*, and *Acetobaceter*, which were ranked in the top four relative abundances, were all negatively correlated with the moisture content, acidity, cellulase activity, and lipase activity. This was due to the presence of diacetyl in the metabolites of *Lactobacillus*, which inhibits many spoilage and pathogenic bacteria. Moreover, it could inhibit the growth of gram-negative bacteria by interfering with the utilization of arginine through the binding protein reaction of gram-negative bacteria with arginine [33].

#### 3.5.2. Correlation Analysis of Fungi at the Genus Level and Physicochemical Indicators

**Figure 11:**
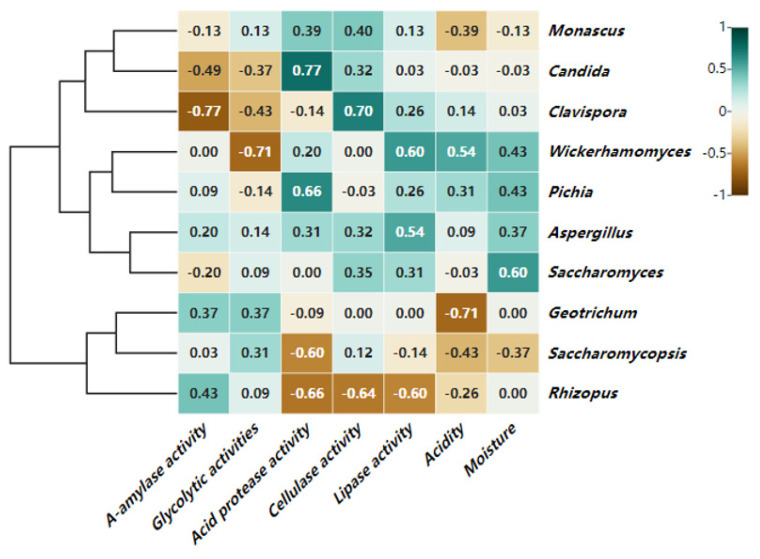
Heat map of correlation between fungi microorganisms and physicochemical indicators based on genus level.

As shown in Figure 11, there were significant interactions between physicochemical factors and most fungal microorganisms, indicating the complexity of the fungal microbial metabolism. Moisture content and acidity were positively correlated with *Pichia* and *Wickerhamomyces*; moisture content, acidity, lipase activity, acid protease activity, and A-amylase activity were negatively correlated with *Saccharomycopsis* and *Rhizopus*; cellulase activity was positively correlated with *Clavwaspora* and *Wickerhamomyces*; and glucoamylase activity was positively correlated with *Rhizopus*. Traditionally, Rhizoctonia solani and others were considered to be the main producers of the saccharification power of macrophytes, which is consistent with the results in this study. The strong positive correlation between acid protease activity and the first dominant bacterium *Saccharomyces* suggests that acid protease production might be associated with yeasts, and *Saccharomycopsis* has been shown to produce acidic proteases capable of degrading large proteins [34].

The microbial composition and environmental factors can have a great influence on the community succession during the fermentation of Baijiu [35], indicating that those two factors are the main drivers that induce the succession of the fermentation from the initial to the end stage.

### 3.6. Qualitative and Quantitative Analysis of VOCs by GC–MS

**Figure 12:**
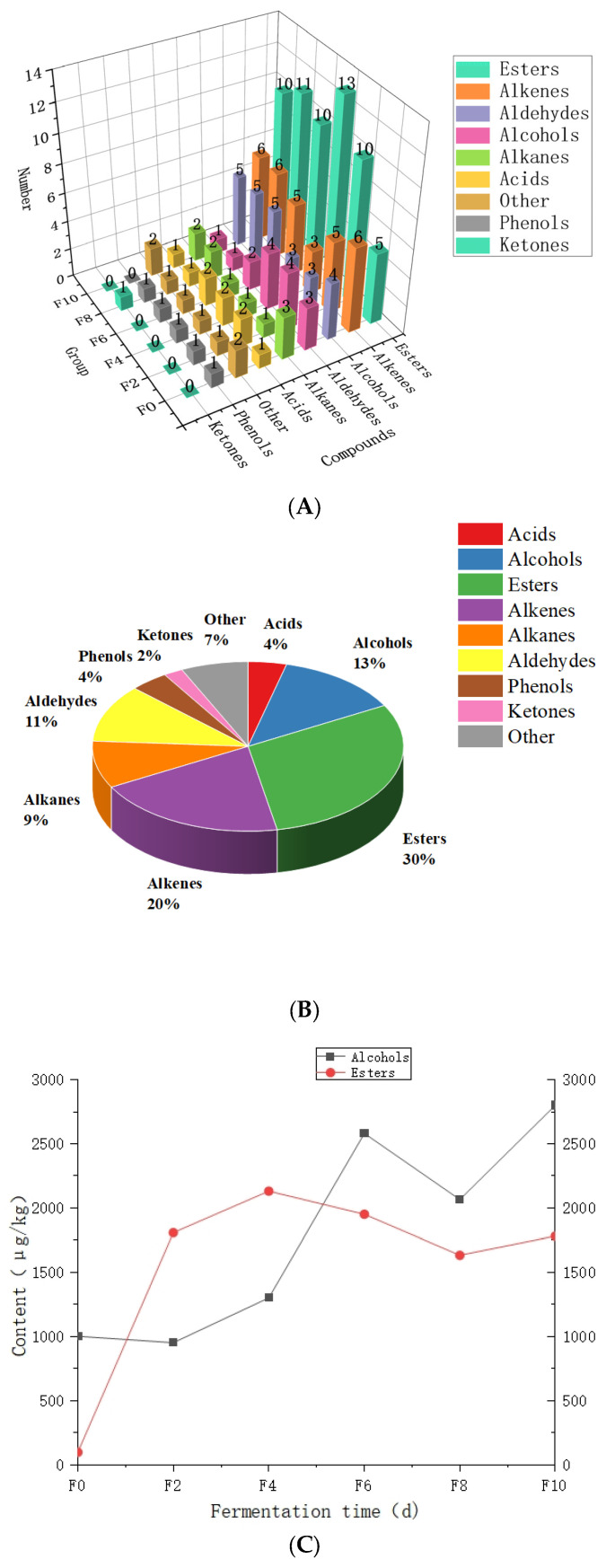
Numbers and percentages of volatile compounds identified in the Tuqu fermentation process of Hunan light-flavor Baijiu: (**A**) numbers of different types of volatile compounds in different steps; (**B**) the percentages of different volatile compounds in the Tuqu fermentation process of Hunan light-flavor Baijiu; (**C**) the changes in the content of alcohols and esters of Hunan Tuqu light-flavor Baijiu.

In this study, Table 2 provides qualitative and quantitative analysis of the identified VOCs. As shown in Figure 12A, a total of 46 VOCs were identified using GC–MS, including aldehydes (5), esters (14), alcohols (6), ketones (1), alkanes (4), phenols (2), acids (2), alkenes (9), and others (3). The relative proportion of these categorized VOCs is illustrated in Figure 12B, with esters (30.4%), alkenes (19.6%) and alcohols (13%) constituting a significant portion, and 63% of the total overall.

Esters were the most abundant class of flavor components in Baijiu, giving it fruity and floral aromas [36]. It was also the most abundant flavor substance detected in the fermented grains of Hunan light-flavor Baijiu, including ethyl lactate, isoamyl acetate, ethyl caprylate, diethyl succinate, ethyl caprate, ethyl phenylacetate, phenethyl acetate, etc. The esters accumulated during fermentation and reached a peak of 2130.6 (μg/kg) on the fourth day of fermentation, increasing from 95.68 (μg/kg) at the beginning of fermentation to 1780.7 (μg/kg) at the end of fermentation, which was 18.6 times higher than that at the beginning of fermentation. Isoamyl acetate, ethyl caprylate, diethyl succinate, and 2-methylbutyl acetate were the most abundant in the fermentation process of the fermented grains, accounting for 79.5% of the esters, with a fruity aroma, and are important aroma and flavor presenting substances in the fermented grains of Hunan light-flavor Baijiu [37].

### 3.7. Critical Volatile Compounds

It is worth noting that not all VOCs detected in the fermented grain samples fermented at different times contribute significantly to the flavor of the fermented grains. An ROAV analysis was conducted to assess the contribution of different volatiles to the fermented grains’ aroma during the fermentation of the fermented grains. The ROAV value indicates the importance of key aroma compounds, with higher scores indicating a greater contribution to the aroma quality of the fermented grains [38]. Among the VOCs analyzed (Table 2), nine key VOCs with ROAV > 1 were identified in the fermented grains: 3-methyl-1-butanol (ROAV = 35.49), 1-octen-3-ol (ROAV = 3.48), 2-methyl-1-butanol (ROAV = 3.95), isoamyl acetate (ROAV = 100), ethyl caprylate (ROAV = 1.24), ethyl caprate (ROAV = 2.30), ethyl caproate (ROAV = 2.76), 2-methylbutyl acetate (ROAV = 8.36), and 1-nonanal (ROAV = 3.78). Isoamyl acetate had the highest ROAV and contributed the most to the flavor of the fermented grains. Its content decreased and then increased, giving the fermented grains a fruity aroma and a banana flavor. 3-methyl-1-butanol was the second most important odor-active substance, contributing to the floral and cocoa flavors of the fermented grains. 1-octen-3-ol, decanal, despite being present in low concentrations, had a high ROAV due to its low odor threshold, and it contributes a mushroom flavor to the fermented grains. 2-methylbutyl acetate provides a fruity and pear aroma to the fermented grains, ethyl caprylate provides a sweet and soft floral and brandy aroma, ethyl caproate provides a brandy and pineapple aroma to the fermented grains, and ethyl caprate provides a brandy and pear aroma to the fermented grains. All these volatile organic compounds enhance the floral and fruity aromas of the fermented grains. VOCs with 0.1 ≤ ROAV < 1 included phenethyl alcohol, ethyl heptanoate, decanal, hexanal, 3-octanone, and 2-pentylfuran, which have a moderating effect on the aroma characteristics of the fermented grains. Phenethyl alcohol increased during fermentation, giving the fermented grains its honey and rose aromas.

## 4. Conclusions

The results demonstrate that the microbial metabolism exhibited significant activity during the pre-fermentation and mid-fermentation stages, which were characterized by a higher water content and the efficient utilization of starch for saccharification. α-amylase and glucoamylase activities displayed a sharp decline, while acid protease, cellulase activity, and lipase activity showed an increase. Additionally, the acidity gradually increased as the fermentation progressed. The microbial structure of Hunan light-flavor Baijiu at different fermentation periods was investigated using high-throughput sequencing technology. A total of fifty bacterial genera and twenty-two fungal genera were identified in this study. Lactobacillus and Saccharomyces were found to be the predominant microorganisms among bacteria and fungi, respectively. Notably, there were substantial variations in the microbial composition and abundance observed across different fermentation periods, highlighting the complexity of the microbial structure during the fermentation stage. The results of a Spearman correlation analysis of physicochemical indicators with fungal and bacterial microorganisms show that *Lactobacillus* and *Saccharomyces* were the dominant genera for bacteria and fungi, respectively, which suggests that these physicochemical indicators drive changes in the flora of *Saccharomyces* and *Lactobacillus*. At the same time, the growth and metabolism of *Saccharomyces* and *Lactobacillus* could also have an important effect on the enzyme activity, ethanol content, and acidity, which are conducive to the formation of the Baijiu flavor. Additionally, a total of 46 volatile flavor compounds were detected via GC-MS in the fermented grains samples, of which esters constituted the major portion, and nine key flavor compounds with an ROAV > 1 were identified throughout the fermentation process. Isoamyl acetate had the highest ROAV and made the greatest contribution to the flavor, enhancing the floral and fruity aroma of the fermented grains. These results provide a data reference and theoretical guidance for the industrial development of light-flavor Baijiu.

## Figures and Tables

**Table 1:** Variations in enzyme activity during the Tuqu fermentation of Hunan light-flavor Baijiu.

Fermentation Days (d)	Alpha Amylase Activity/(U/g)	Acid Protease Activity/(U/g)	Glucoamylase Activity/(U/g)	Cellulase Activity/(U/g)	Lipase Activity/(U/g)
0	28.1	7.6	1027.1	118.9	31.1
2	6.5	30.6	809.8	473.8	34.3
4	4.2	25.7	786.2	607.7	34.8
6	3.5	20.5	691.8	603.1	33.5
8	4.9	14.7	732.5	607.1	35.1
10	3.8	16.7	766.7	668.7	36.4

**Table 2:** VOCs during the Tuqu fermentation of Hunan light-flavor Baijiu.

NO	Compounds	CAS	RI a/RI b	Identification c	F0	F2	F4	F6	F8	F10	Thresholds (μg/kg)	F0	F2	F4	F6	F8	F10
Acids																	
a1	2-Hydroxypropan	50–21-5	838/835	MS, RI	n.d.	5.86 ± 0.46 C	15.90 ± 1.19 A	11.81 ± 0.94 B	n.d.	n.d.	n.f.	-	-	-	-	-	-
a2	2-amino-4-methylbenzoic acid	2305-36-4	949/925	MS, RI	159.79 ± 19.92	240.52 ± 26.93 C	244.87 ± 16.50 AB	224.45 ± 9.42 AB	266.98 ± 10.69 A	189.91 ± 58.27 BC	n.f.	-	-	-	-	-	-
Alcohols																	
a3	3-Methyl-1-butanol	123-51-3	736/740	MS, RI	698.71 ± 23.34 B	296.73 ± 50.96 D	472.78 ± 20.98 C	459.49 ± 17.67 C	1219.52 ± 136.97 A	502.99 ± 53.32 C	4	35.49	2.44	5.29	6.85	19.32	3.52
a4	1-Octen-3-ol	3391-86-4	980/981	MS, RI	25.72 ± 8.43 A	15.91 ± 3.06 B	24.61 ± 2.56 A	15.65 ± 0.50 B	18.56 ± 2.31 B	15.30 ± 1.24 B	1.5	3.48	0.35	0.73	0.62	0.78	0.29
a5	2-Ethylhexan-1-ol	104-76-7	1030/1032	MS, RI	3.00 ± 0.40 A	n.d.	n.d.	n.d.	n.d.	n.d.		-	-	-	-	-	-
a6	Phenethyl alcohol	60-12.8	1116/1117	MS, RI	273.40 ± 1.71 E	637.84 ± 52.93 D	802.88 ± 84.86 B	1031.92 ± 99.92 A	485.74 ± 19.76 D	736.40 ± 71.82 BC	564.23	0.10	0.04	0.06	0.11	0.05	0.04
a7	2-Methyl-1-butanol	137-32-6	739/738	MS, RI	n.d.	n.d.	n.d.	1052.98 ± 17.40 B	328.97 ± 7.45 C	1513.12 ± 99.28 A	15.9	0.00	0.00	0.00	3.95	1.31	2.66
a8	1-Octanol	111-87-5	1071/1074	MS, RI	n.d.	n.d.	n.d.	20.65 ± 2.36 B	12.74 ± 0.82 C	32.64 ± 2.16 A	125.8	0.00	0.00	0.00	0.01	0.01	0.01
Esters																	
a9	Ethyl lactate	97-64-3	815/812	MS, RI	7.25 ± 0.17 E	13.30 ± 0.96 D	54.60 ± 0.90 B	69.38 ± 3.46 A	44.29 ± 1.99 B	34.78 ± 4.87 C	n.f.	-	-	-	-	-	-
a10	Isoamyl acetate	123-92-2	876/876	MS, RI	73.83 ± 6.55 D	455.61 ± 38.73 AB	335.35 ± 22.99 B	251.52 ± 19.00 C	236.75 ± 7.32 C	536.37 ± 26.61 A	0.15	100.00	100.00	100.00	100.00	100.00	100.00
a11	Ethyl caprylate	106-32-1	1196/1200	MS, RI	7.15 ± 0.91 C	352.63 ± 11.79 A	369.77 ± 33.46 A	401.26 ± 50.43 A	239.56 ± 31.40 B	369.52 ± 17.22 A	19.3	0.08	0.60	0.86	1.24	0.79	0.54
a12	Diethyl succinate	123-25-1	1182/1186	MS, RI	2.75 ± 0.46 E	513.20 ± 47.83 B	586.85 ± 23.91 A	500.78 ± 25.07 B	248.46 ± 62.24 D	379.88 ± 10.56 C	n.f.	-	-	-	-	-	-
a13	Ethyl caprate	110-38-3	1396/1401	MS, RI	4.70 ± 0.49 D	85.34 ± 3.71 C	126.78 ± 2.47 b	192.61 ± 15.70 A	97.32 ± 5.20 C	92.56 ± 5.18 C	5	0.19	0.56	1.13	2.30	1.23	0.52
a14	Ethyl phenylacetate	101-97-3	1246/1248	MS, RI	n.d.	5.99 ± 0.39 A	5.33 ± 0.41 B	5.93 ± 1.76 A	6.70 ± 0.16 A	6.87 ± 0.19 A	n.f.	-	-	-	-	-	-
a15	Phenethyl acetate	103-45-7	1258/1259	MS, RI	n.d.	58.08 ± 9.51 B	71.15 ± 3.07 A	79.47 ± 3.86 A	72.91 ± 4.07 A	52.40 ± 1.42 A	249.59	0.00	0.01	0.01	0.02	0.02	0.01
a16	Ethyl caproate	123-66-0	1000/1004	MS, RI	n.d.	311.90 ± 23.51 A	308.75 ± 27.45 A	n.d.	n.d.	n.d.	5	0.00	2.05	2.76	0.00	0.00	0.00
a17	Ethyl nonanoate	123-29-5	1296/1299	MS, RI	n.d.	7.24 ± 1.25 B	9.38 ± 1.21 A	n.d.	n.d.	n.d.	n.f.	-	-	-	-	-	-
a18	Ethyl laurate	106-33-2	1595/1605	MS, RI	n.d.	4.08 ± 0.82 BC	5.03 ± 0.90 ABC	6.65 ± 1.30 A	5.28 ± 1.55 AB	3.38 ± 0.17 C	n.f.	-	-	-	-	-	-
a19	2-Methylbutyl acetate	624-41-9	880/880	MS, RI	n.d.	n.d.	233.73 ± 12.48 D	422.28 ± 18.13 B	659.90 ± 28.35 A	292.66 ± 20.03 C	5	0.00	0.00	2.09	5.04	8.36	1.64
a20	2-hydroxy-4-methylvalerate ethyl ester	10348-47-7	1060/1061	MS, RI	n.d.	n.d.	16.76 ± 0.53 B	20.74 ± 1.93 A	12.74 ± 0.82 C	12.28 ± 2.09 C	n.f.	-	-	-	-	-	-
a21	Ethyl heptanoate	106-30-9	1097/1105	MS, RI	n.d.	n.d.	7.12 ± 0.31 a	n.d.	n.d.	n.d.	1.9	0.00	0.00	0.17	0.00	0.00	0.00
a22	Hexyl acetate	142-92-7	1011/1016	MS, RI	n.d.	n.d.	n.d.	n.d.	8.46 ± 0.20 A	n.d.	n.f.	-	-	-	-	-	-
Alkenes																	
a23	1-Nonene	124-11-8	889/879	MS, RI	42.09 ± 2.52 A	n.d.	n.d.	n.d.	n.d.	n.d.	n.f.	-	-	-	-	-	-
a24	3-Methyl-1-heptene	4810/9/7	1075/1071	MS, RI	14.92 ± 0.50 B	13.85 ± 0.39 B	10.50 ± 0.35 C	16.04 ± 1.50 B	23.50 ± 3.01 B	440.40 ± 12.97 A	n.f.	-	-	-	-	-	-
a25	1-Dodecene	112-41-4	1190/1192	MS, RI	6.79 ± 0.41 B	4.94 ± 0.71 B	n.d.	n.d.	11.52 ± 3.59 A	10.18 ± 0.77 A	n.f.	-	-	-	-	-	-
a26	1, 2, 3, 3 cyclopentadiene	65372-78-3	1386/1387	MS, RI	2.27 ± 0.99 C	3.21 ± 0.29 C	5.53 ± 0.32 B	7.88 ± 1.29 A	n.d.	n.d.	n.f.	-	-	-	-	-	-
a27	1-Tetradecene	1120-36-1	1392/1394	MS, RI	3.49 ± 0.78 B	n.d.	n.d.	4.83 ± 0.17 A	4.16 ± 0.71 AB	2.45 ± 0.67 C	n.f.	-	-	-	-	-	-
a28	(Z)-3-Un.d.ecene	821-97-6	1086/1074	MS, RI	n.d.	20.78 ± 5.27 A	n.d.	n.d.	n.d.	n.d.	n.f.	-	-	-	-	-	-
a29	l-Caryophyllene	87-44-5	1419/1421	MS, RI	11.40 ± 0.42 BC	11.83 ± 1.19 BC	14.53 ± 1.70 B	25.64 ± 3.57 A	8.89 ± 1.16 C	9.30 ± 1.01 C	64	0.04	0.01	0.01	0.02	0.01	0.00
a30	4-Methoxystyrene	637-69-4	1156/1151	MS, RI	n.d.	n.d.	n.d.	6.34 ± 1.15 B	23.36 ± 0.25 A	3.53 ± 1.09 C	n.f.	-	-	-	-	-	-
a31	1, 2, 3, 3 cyclopentadiene	65372-78-3	1386/1386	MS, RI	n.d.	n.d.	n.d.	n.d.	3.45 ± 0.82 A	3.01 ± 0.78 A	n.f.	-	-	-	-	-	-
Alkanes																	
a32	Cis-1, 2-dimethylcyclopentane	1192-18-3	723/724	MS, RI	715.98 ± 33.87 C	937 ± 83.22 A	814.94 ± 58.83 B	664.03 ± 58.58 C	656.27 ± 39.86 C	826.49 ± 36.56 B	n.f.	-	-	-	-	-	-
a33	2, 6, 11-Trimethyldodecane	31295-56-4	1275/1281	MS, RI	4.04 ± 0.59 A	n.d.	n.d.	n.d.	n.d.	n.d.	n.f.	-	-	-	-	-	-
a34	Tridecane	629-50-5	1300/1301	MS, RI	4.94 ± 0.77 A	n.d.	n.d.	n.d.	n.d.	n.d.	n.f.	-	-	-	-	-	-
a35	4, 6-Dimethyldodecane	61141-72-8	1325/1327	MS, RI	n.d.	n.d.	n.d.	n.d.	6.88 ± 0.59 A	5.63 ± 0.79 B	n.f.	-	-	-	-	-	-
Aldehydes											n.f.						
a36	Phenylacetaldehyde	122-78-1	1045/1046	MS, RI	4.63 ± 0.23 C	6.75 ± 0.64 B	7.99 ± 1.25 A	8.14 ± 0.88 A	n.d.	n.d.	6.3	0.15	0.04	0.06	0.08	0.00	0.00
a37	Decanal	112-31-2	1206/1208	MS, RI	1.34 ± 0.13 CD	4.23 ± 0.34 C	3.29 ± 0.96 CD	3.11 ± 0.34 CD	10.04 ± 2.27 A	7.81 ± 0.56 B	3	0.09	0.05	0.05	0.06	0.21	0.07
a38	Hexanal	66-25-1	800/803	MS, RI	n.d.	49.69 ± 4.45 A	n.d.	n.d.	n.d.	n.d.	5	0.00	0.33	0.00	0.00	0.00	0.00
a39	1-Nonanal	124-19-6	1104/1106	MS, RI	7.51 ± 1.75 C	51.91 ± 11.88 B	92.99 ± 7.91 A	n.d.	n.d.	n.d.	1.1	1.39	1.55	3.78	0.00	0.00	0.00
a40	2, 5-Dimethylbenzaldehyde	5779-94-2	1208/1217	MS, RI	n.d.	n.d.	6.58 ± 0.38 A	n.d.	n.d.	n.d.	n.f.	-	-	-	-	-	-
Phenols																	
a41	2, 4-Di-tert-butylphenol	96-76-4	1519/1520	MS, RI	3.55 ± 0.40 A	n.d.	n.d.	n.d.	n.d.	n.d.	n.f.	-	-	-	-	-	-
a42	4-Ethylguaiacol	2785-89-9	1282/1281	MS, RI	n.d.	13.13 ± 1.55 A	12.90 ± 0.75 A	12.39 ± 0.62 A	13.46 ± 0.62 A	n.d.	89.25	0.00	0.00	0.01	0.01	0.01	0.00
Ketones																	
a43	3-Octanone	106-68-3	986/989	MS, RI	n.d.	n.d.	n.d.	n.d.	35.02 ± 1.69 A	n.d.	21.4	0.00	0.00	0.00	0.00	0.10	0.00
Other																	
a44	Naphthalene	91-20-3	1182/1182	MS, RI	8.46 ± 0.45 A	n.d.	n.d.	n.d.	n.d.	n.d.	n.f.	-	-	-	-	-	-
a45	Benzylhydrazine	555-96-4	1118/1122	MS, RI	69.00 ± 3.06 C	319.93 ± 13.71 B	37.13 ± 2.48 D	46.18 ± 3.53 D	352.31 ± 23.38 A	46.57 ± 4.17 D	n.f.	-	-	-	-	-	-
a46	2-Pentylfuran	3777-69-3	993/992	MS, RI	n.d.	n.d.	n.d.	n.d.	n.d.	44.27 ± 5.13 A	5.8	0.00	0.00	0.00	0.00	0.00	0.21

^a^: retention index of compounds on HP-5MS; ^b^: retention index of compounds in references; ^c^: “MS”: mass spectrum comparison using NIST17 library; A–E: different letters represent significant differences. “RI”: retention index in agreement with the literature value; ‘n.f.’: threshold was not found in the literature; ‘n.d.’: data were not detected in the sample. The threshold of volatile compounds in water refers to the literatures [13].

## Data Availability

The original contributions presented in the study are included in the article; further inquiries can be directed to the corresponding author.
